# Advances in the prevention of invasive pneumococcal disease in childhood

**DOI:** 10.36416/1806-3756/e20260210

**Published:** 2026-05-22

**Authors:** Julia Mundstock Noethen, Maria Paula de Carli Hanel, Amanda Bendorovicz Trevisan Dorneles, Leonardo Araujo Pinto, Marcelo C Scotta

**Affiliations:** 1. Grupo de Pesquisa em Epidemiologia e Genética das Doenças Respiratórias da Infância, Escola de Medicina, Pontifícia Universidade Católica do Rio Grande do Sul - PUCRS - Porto Alegre (RS) Brasil.; 2. Programa de Pós-Graduação em Medicina - Pediatria, Escola de Medicina, Pontifícia Universidade Católica do Rio Grande do Sul - PUCRS - Porto Alegre (RS) Brasil.

## INTRODUCTION

Infection caused by *Streptococcus pneumoniae* remains one of the leading causes of pneumonia, meningitis, and sepsis in children under five years of age, with a significant impact on global morbidity and mortality. The introduction of pneumococcal conjugate vaccines (PCVs) has substantially altered the epidemiological landscape of invasive pneumococcal disease (IPD), leading to a marked reduction in hospitalizations in the years following their implementation. In Brazil, the incorporation of PCV10 into the *Programa Nacional de Imunizações* (PNI, National Immunization Program) in 2010 was associated with a significant decline in hospitalization rates for childhood pneumonia, demonstrating both direct and indirect effects of the vaccination strategy.^1^

However, pneumococcal dynamics are characterized by high serotypic diversity and adaptive capacity. Following the implementation of PCV10, the phenomenon of serotype replacement has been observed, with a progressive increase in non-vaccine serotypes. Surveillance data from Brazil demonstrate a consistent shift in the distribution of serotypes that cause IPD over the past decade.^1^ ^,^ ^2^ Among the emerging serotypes, 19A stands out, having become the leading serotype associated with IPD in Brazilian children in the post-PCV10 era. Since 2014, it has accounted for 28.2-44.6% of cases in children under five years of age. In addition to its high prevalence, it is frequently associated with more severe clinical presentations and increased antimicrobial resistance, further enhancing its clinical and epidemiological relevance. In parallel, serotypes such as 3 and 6C have also shown increasing contributions to the residual disease burden in children younger than five years.

## EVOLUTION OF PNEUMOCOCCAL VACCINES

PCV10 was introduced into the Brazilian PNI in 2010, while PCV13 became available in the private sector in the same year. Only in 2019 was PCV13 incorporated into the PNI and the *Centros de Referência para Imunobiológicos Especiais* (CRIE, Special Immunobiological Referral Centers), being offered to children at higher risk of IPD, particularly those with (primary or secondary) immunodeficiencies, asplenia or sickle cell disease, cystic fibrosis, solid organ transplant recipients, as well as conditions such as cerebrospinal fluid leaks and chronic disabling neurological diseases. More recently, PCV15 and PCV20 were introduced into the Brazilian private market in 2023 and are not yet available through the PNI/CRIE.^3^

PCV10 and PCV13 differ in the number of included serotypes, polysaccharide concentration, conjugation methods, and carrier proteins. In PCV10, eight serotypes are conjugated to protein D, a surface lipoprotein of non-typeable *Haemophilus influenzae* , while the remaining two serotypes are conjugated to tetanus and diphtheria toxoids. In contrast, all serotypes in PCV13 are conjugated to the carrier protein CRM197, a non-toxic variant of diphtheria toxin derived from *Corynebacterium diphtheriae*, and additionally include serotypes 3, 6A, and 19A. The more recent vaccines (PCV15 and PCV20) also use CRM197 as a carrier protein, expanding serotype coverage: PCV15 adds serotypes 22F and 33F, while PCV20 further includes serotypes 8, 10A, 11A, 12F, and 15B.

The transition toward higher-valency vaccines aims to address existing gaps in protection through more robust conjugation technologies.^2^ This technical distinction is clinically relevant, as CRM197 has been shown to induce a stronger immunological memory response, which is essential for the control of persistent pathogens. An example is PCV15, which employs optimized conjugation techniques to enhance immunogenicity against serotype 3 when compared with PCV13.^2^ ^,^ ^5^

## FUTURE DIRECTIONS IN PNEUMOCOCCAL PREVENTION

The inclusion of these vaccines in the Brazilian context aims to increase coverage against serotypes that have become more prevalent due to their lack of coverage by PCV10. According to data from the Adolfo Lutz Institute, among children under one year of age in 2025, and considering serotypes exclusively covered by each PCV, PCV13 covered 53.6% of circulating strains, whereas PCV10 covered 2.4%. These findings highlight the need for continued advances in the prevention of IPD.^2^ ^,^ ^4^

In accordance with guidelines from the Brazilian Society of Pediatrics, in conjunction with the Brazilian Society of Immunizations and the Brazilian Society of Infectious Diseases, higher-valency pneumococcal vaccines are preferentially recommended for children under five years of age in comparison with the vaccine currently available in the PNI. In the updated 2025/2026 immunization schedule, the Brazilian Society of Pediatrics recommends, whenever possible, the use of PCV13, PCV15, or PCV20 in a 3+1 schedule (at 2, 4, and 6 months of age, followed by a booster dose between 12 and 15 months).^5^

[Table t1]

**Table 1 t1:** Characteristics and coverage of major pneumococcal vaccines available :

Vaccine	Serotypes	Carrier protein (adjuvant)	Commercial name (Manufacturer)
PCV10	1, 4, 5, 6B, 7F, 9V, 14, 18C, 19F, 23F	Protein D from non-typeable *Haemophilus influenzae*, tetanus toxoid, and diphtheria toxoid	Synflorix® (GSK)
PCV13	1, 3, 4, 5, 6A, 6B, 7F, 9V, 14, 18C, 19A, 19F, 23F	CRM197 protein	Prevenar 13® (Pfizer)
PCV15	1, 3, 4, 5, 6A, 6B, 7F, 9V, 14, 18C, 19A, 19F, 22F, 23F, 33F	CRM197 protein	VaxNeuvance® (MSD)
PCV20	1, 3, 4, 5, 6A, 6B, 7F, 8, 9V, 10A, 11A, 12F, 14, 15B, 18C, 19A, 19F, 22F, 23F, 33F	CRM197 protein	Prevenar 20® (Pfizer)

Currently, a protection gap driven by socioeconomic access is evident: while scientific societies preferentially recommend higher-valency vaccines, universal availability within the PNI remains limited to PCV10 or in a slow transition to higher-valency vaccines. This disparity results in unequal epidemiological risk, with children dependent on the public health care system being more exposed to emerging serotypes.^1^ ^,^ ^2^ ^,^ ^5^ To mitigate this scenario while the full implementation of those vaccines into the PNI has not yet been achieved, clinical strategies should focus on vaccine interchangeability and schedule complementation. Of note, as PCV10 is based on a different carrier protein from those in higher-valency vaccines, it is necessary to complete a new schedule with PCV13, PCV15, or PCV20 in individuals previously immunized with PCV10. However, true population-level effectiveness ultimately depends on the universalization of these technologies, transforming scientific advances into a tool for social equity that reaches the entire pediatric population, regardless of their socioeconomic status.^3^ ^,^ ^5^

## CONCLUSION

The future of pneumococcal prevention in Brazil depends on the timely alignment of public health policy with current biotechnological and epidemiological realities. The transition to higher-valency vaccines within the PNI is not merely a technical update, but it is also a strategic necessity to address the adaptive capacity of *S. pneumoniae* and to mitigate antimicrobial resistance. Continuous surveillance and democratization of access to broader-coverage vaccines are essential pillars to ensure that biotechnological innovation translates into a meaningful, sustained, and equitable reduction in childhood morbidity and mortality across the country.^1^ ^,^ ^2^
